# Investigation on the Curvature and Stress Distribution of Laminates Based on an Analytic Solution and FE Simulation

**DOI:** 10.3390/ma15186458

**Published:** 2022-09-17

**Authors:** Chao Liu, Anne Günther, Yuanbin Deng, Anke Kaletsch, Mathias Herrmann, Christoph Broeckmann

**Affiliations:** 1 Institute for Materials Applications in Mechanical Engineering (IWM), RWTH Aachen, 52062 Aachen, Germany; 2 Fraunhofer Institute for Ceramic Technologies and Systems IKTS, 01277 Dresden, Germany

**Keywords:** metal-ceramic laminates, finite element simulation, analytic solution, normalized curvature, stress distribution

## Abstract

The potential combinations of favorable properties give metal–ceramic laminates (MCLs) a high degree of application flexibility. However, the different thermal expansion coefficients (CTEs) and shrinkage rates of the metals and ceramics during the co-sintering process often lead to large internal stresses that cause undesired deformation or even production failures. In practice, the identification of manufacturable MCLs relies on the “trial and error” principle, which usually requires a long development period. Therefore, there is a great demand for analytic and numerical methods that allow the prediction of the deformation and manufacturability of MCLs during the co-sintering process. The main objective of this study is to investigate the curvature and stress distribution in the MCLs (steel 17-4PH/ ceramic 3Y-TZP) based on the analytic solution and finite element (FE) simulation. To achieve this, the Young’s moduli (E) and shear moduli (G) at high temperatures and the CTEs of both materials were measured. In addition, the curvatures and stress distributions of the two-layer and three-layer laminates were obtained based on the analytic method and FE simulation, which were in very good agreement. Furthermore, the influence of the CTE, Young’s modulus, height ratio, and interface on the curvature were studied. The results showed that the CTE and height ratio have a higher influence on the curvature in comparison to the Young’s modulus. The interface prevents the curvature significantly by assuming it to be a cohesive surface in the FE simulation. This provides hints to avoid delamination during the manufacturing process.

## 1. Introduction

Ceramic materials are used in many different application fields due to their high thermal and chemical stability, electrical resistivity, and high hardness and wear resistance [1] (pp. 9–17). Typical examples include wear parts and bearings, electrical insulation, cutting tools, and refractory materials. Recently, there has been an increasing demand for multi-functional materials and components, which usually require lamination and the co-sintering of different components [2]. For instance, in order to combine local electrical conductivity with insulating properties, the combination of metallic components with ceramics is necessary. In addition, the brittle fracture behavior can be positively influenced to achieve high toughness and damage tolerance through grading or the combination of different components [3] (p. 733). More specifically, metal–ceramic laminates (MCLs) exhibit increased stress resistance and reliability in comparison to purely brittle ceramic materials, since the metallic component has the ability to deform plastically and deflect or even stop the cracks that occur and propagate in the ceramic component [3] (p. 734).

Until now, such laminates have essentially been produced following the procedure of tape casting, lamination, debinding, and co-sintering. By using green tapes with different compositions, composites with different materials and property gradients can be produced during the final debinding and co-sintering process [4,5]. The combinations of a wide range of favorable properties give the metal–ceramic composites a high degree of flexibility. This process enables the use of metal-ceramic composites in previously unexplored applications, because the required functional areas of the laminates can be assigned to components with appropriate properties and can be designed specifically for the application [3] (p. 734).

Nevertheless, due to differences in chemical composition and particle morphology, the materials combined in the laminate usually exhibit different sintering shrinkage rates, coefficients of thermal expansion (CTE), and elastic moduli. Apart from the different material properties, the height ratio and interface will also affect the curvature and stress distribution of the laminates significantly. All of these factors will result in the mismatch of the transverse strain during sintering, leading to internal stresses that can cause deformation or even early failure of the component [3,6,7,8,9]. Therefore, a major challenge lies in the identification of compatible materials for which an existing mismatch of the strain during co-sintering does not lead to undesired deformations or production defects. In practice, the identification of a defect-free, near-net-shape layer composite design relies frequently on the “trial and error” principle. For instance, Dourandish et al. [10] have investigated the possibility of pressureless sintering for compacted 3Y-TZP (two particle sizes) with three different stainless steels (17-4PH, 316L, and 420 SS) under various sintering atmospheres (H_2_, Ar, Ar+H_2_, and vacuum). They found that the mismatch strain between 3Y-TZP and SS during co-sintering can lead to bond cracking and joint failure. The joining of 3Y-TZP (150 nm) to 420 SS under vacuum conditions appears to be most successful among the investigated combinations. Another “trial and error” investigation was carried out by Slawik et al. [11], who used four different compositions of metallic slurries for tape casting and subsequent co-sintering with 3Y-TZP to obtain symmetric and asymmetric MCLs. The spherical metallic powder was milled in advance to increase its specific surface area so that a larger sintering strain could be obtained [12]. It was found that the relative density of the zirconia layer in the MCLs was reduced due to a less shrinkage in the metallic layer. Moreover, cracks occur in the ceramic layer due to mismatch in the strain rate. Nevertheless, the manufacturing of defect-free MCLs with dense ceramic layers is still achievable by adapting the sintering behavior of metallic powder.

The “trial and error” approach usually results in a high failure ratio and requires a long development period. Consequently, there is a high demand for numerical and simulation methods that allow reliable predictions of the manufacturability, as well as the deformation during the co-sintering process. The analytical solution for the curvature and stress distribution due to different the CTEs and material stiffness levels of bi-material laminates was derived by Timoshenko [13] almost one century ago. This theory assumes an identical curvature of a bi-metal strip, and the results were obtained from force and strain equilibrium results. This analytical solution is not only capable of analyzing a one-dimension beam structure (i.e., beam theory), but can also be applied to plate geometry by assuming an equ-biaxial plane stress state and including the Poisson’s ratio in the Young’s modulus [13].

The Timoshenko’ beam (TB) theory enjoys the merits of a simple implementation approach and allows an analysis of the stress distribution throughout the whole thickness. Thus, this theory has been widely used to predict the thermal residual stress and curvature in different combinations of bi-layer materials. Swain [14] successfully used the TB theory to investigate the causes of residual stress development in dental ceramics, which consist of a ceramic coping material (alumina, zirconia, glass ceramic, etc.) and porcelain. By considering both the CTE mismatch stresses and tempering stresses that arise from the large temperature gradient due to rapid cooling and poor thermal conductivity, he proposed several potential approaches to avoid the chipping of dental structures. With the help of TB theory, Hsueh et al. [15,16] derived the close-form solutions for bilayer dental ceramics subjected to both thermal stresses and biaxial flexure tests. These solutions were validated using a finite element (FE) simulation and were subsequently applied to study the layer thickness effect on the strength of bilayer dental ceramics. Apart from bilayer dental ceramics, the TB theory has also been used to analyze the residual stresses in epoxy–steel laminates by Yu et al. [17] and Jumbo et al. [18]. A good agreement of the curvature values between the experimental results and analytic solution was demonstrated.

Cai et al. [7] broadened the application areas of TB theory and brought it to the world of viscous sintering using the well-known viscous–elastic (VE) analogy. The viscous relations were obtained by replacing the Young’s modulus, mismatch strain, and curvature with the uniaxial viscosity, mismatch strain rate, and curvature rate, respectively. Based on these relations, Cai et al. [7] and Green et al. [19] successfully predicted the curvature of an alumina–zirconia asymmetric laminate after obtaining the uniaxial viscosity via cyclic loading dilatometry [20]. Later on, Ravi and Green [21] demonstrated that the methodology that was originally developed by Cai et al. [7] for layered structures can also be applied to predict the rate of normalized curvature of the bilayer structures made of the same material (alumina) but with different green densities. Furthermore, Chang et al. [22] and Reynier [23] used this theory in the cofiring of bilayer samples for solid oxide fuel cell (SOFC) applications. The calculated curvatures from the analytic model used by Cai et al. [7] are in good quantitative agreement with the experimental observations. Based on this, they concluded that this model could serve as useful methodology for optimizing the design of SOFC products. This model was also used by Ollagnier et al [24] to help understand the densification mechanisms in the constrained sintering process of an asymmetric glass ceramic laminate. With the help of this analytic model, Ni et al. [25] was able to predict the normalized curvature rate developed during co-firing of bilayer laminates. Meanwhile, Wu et al. [26] managed to reduce mismatches of the shrinkage rate and avoid sintering cracks. At the end, a crack-free specimen made of functionally graded materials was obtained.

Despite the wide and successful applications of the TB theory and analytic model in predicting the curvature and residual stress, they have several restrictions and must be applied with caution:

- They assume that the material properties (CTE, Young’s modulus and Poisson’s ratio) are uniform and constant;
- They ignore the friction, assume a perfect tie between layers, and cannot be applied when delamination occurs;
- They assume an equ-biaxial plane stress state, which indicates that they are only suitable for thin plates where the plane dimensions are significantly larger than the thickness;
- They are not able to consider the temperature gradient inside laminates, which would be very critical if the component was large with low thermal conductivity;
- They are (at the current stage) not capable of considering the plastic strain, hardening, creep, and transformation plasticity that commonly occur in steel layers during the cooling down process.

To this end, the well-established and continuum-mechanic-based FE simulation is more suitable to deal with 3D complex geometries accompanied by various material behaviors and boundary conditions. In this study, we exclusively focus on the cooling down step of the co-sintering process. The two-layer and three-layer MCLs are assumed to be free of stress and curvature at the beginning of the cooling down step. The objective is, thus, to investigate the influence of the different Young’s moduli, height ratios, and CTEs of metals and ceramics and their interface on the curvature of two-layer MCLs during the cooling down step based on an analytic solution and FE simulation. In addition, we aim to study the stress distribution of two-layer and three-layer MCLs. With this information obtained, we attempt to provide hints to decrease the curvature and avoid delamination during the manufacturing process.

## 2. Materials and Methods

### 2.1. Materials

**Figure 1:**
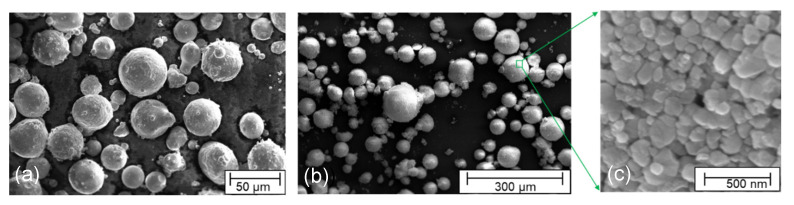
SEM images of (**a**) 17-4PH particles, (**b**) 3Y-TZP granulates, and (**c**) 3Y-TZP particles.

The two materials under investigation are 17-4PH martensitic precipitation-hardened stainless steel and 3Y-TZP 3 mol% yttria-stabilized zirconia (TZ-3Y-SE), produced by Sandvik Osprey, Ltd. (Neath, UK), and Tosoh (Tokyo, Japan), respectively. Both materials are commercially available in the market. The middle particle size $d_{50}$ of 17-4PH and average particle size $\bar{d}$ of TZ-3Y-SE are respectively 17~19 μm and 90 nm. These two materials were chosen to manufacture MCLs because they have similar CTE and shrinkage rates [27]. The particle size and shape were captured under a high-resolution SEM device (FEI Helios NanoLab G3 CX, ThermoFisher, Waltham, MA, USA), as shown in Figure 1. Both the 17-4PH particle and 3Y-TZP granulate are generally spherical. In addition, the 3Y-TZP particles have no apparent preferential length direction. This indicates whether the initial relative density distribution is homogeneous, and if no temperature gradient occurs during free sintering, isotropic sintering shrinkage is expected.

### 2.2. Measurement of E&G Moduli at High Temperature

In order to investigate the influence of the Young’s moduli (E) and shear moduli (G) of both materials on the curvature of two-layer MCLs, the E&G moduli of the 17-4PH and 3Y-TZP at high temperatures needed to be measured. The 17-4PH specimens were produced by tape casting, lamination, debinding, and sintering processes. Due to their sensitivity to oxygen (oxidation), the steel specimens were sintered in 80% argon (Ar)/20% hydrogen (H_2_) at 1350 °C for 2 h (OHV 250/300-1900V, MUT, Wedel, Germany). After sintering, the specimens had dimensions of about 70 × 7 × 3 mm^3^ and a relative density of around 0.97 according to the Archimedes method. The measurement of the E&G moduli of 17-4PH at high temperature was performed in a vacuum using a resonant beam apparatus according to EN 15335:2007 [28]. The E&G moduli were calculated from the resonant frequencies, dimensions, and density of the test specimen.

The 3Y-TZP specimens were uniaxially pressed at 40 MPa and subsequently cold isostatically pressed (VO 19273, EPSI, Temse, Belgium) at 400 MPa for 5 min to achieve a homogeneous density distribution. The specimens were subsequently sintered in air at 1350 °C for 2 h (Denta-Star M2, Thermo-Star, Aachen, Germany). After sintering, the ceramic specimens had dimensions of about 70 × 15 × 3 mm^3^ and a relative density of around 0.99. The measurement was carried out based on the impulse excitation method according to ASTM E1876-21 [29]. The E&G moduli were respectively calculated after capturing the longitudinal and torsional resonant frequencies.

**Figure 2:**
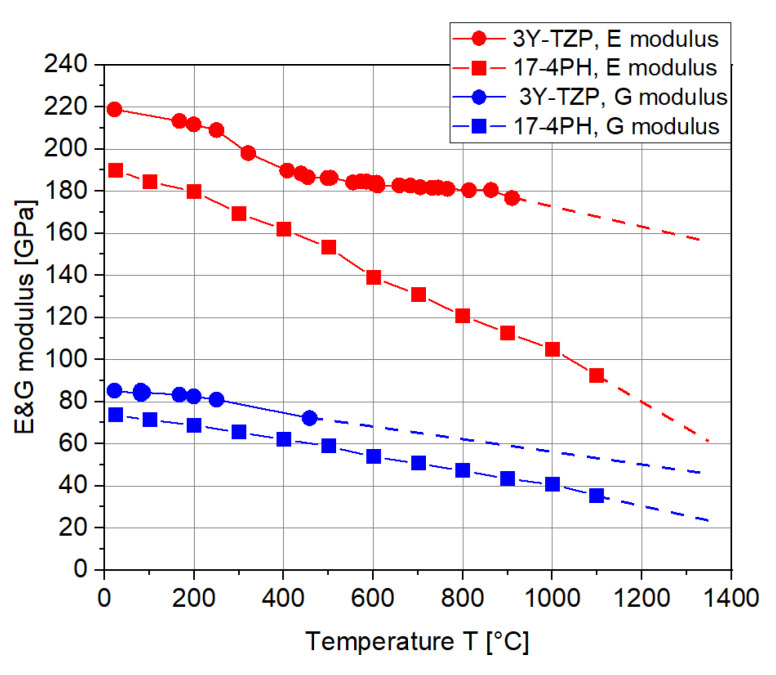
Young’s (E) and shear (G) moduli of 17-4PH and 3Y-TZP at high temperatures.

**Figure 3:**
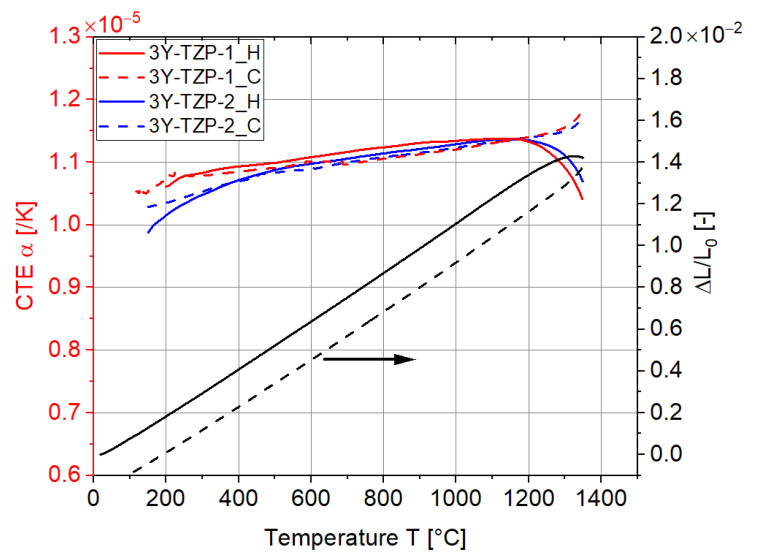
Length changes in specimen 1 and technical CTE results for 3Y-TZP during both heating (H) and cooling (C) steps.

Figure 2 illustrates the temperature-dependent E&G moduli of both materials. Apparently, the E&G moduli of both materials decrease with similar tendencies at high temperatures. At high temperatures, the signal is always difficult to capture, especially in the case of the G modulus of 3Y-TZP. In this situation, all of the values were linearly extrapolated up to the temperature of 1350 °C (dash line in Figure 3) so as to be used in the analytical solution and FE simulation.

### 2.3. Measurement of Coefficient of Thermal Expansion (CTE)

The CTEs of 17-4PH and 3Y-TZP were measured in argon using the horizontal dilatometer (DIL402C, NETZSCH, Selb, Germany). The preparation procedures of the specimens for the measurement of CTE are identical to the specimens for the measurement of the E&G moduli. After sintering, the rectangular 17-4PH and rod 3Y-TZP specimens had dimensions of about 20 × 4 × 3 mm^3^ and Ø5 × 20 mm^3^, respectively.

During each measurement, the specimen was heated at 5 K/min, held for 10 min, and then cooled down to room temperature at −5 K/min. The results of the length change $\frac{\Delta L}{L_{0}}$ and the corresponding technical CTE α of 3Y-TZP are shown in Figure 3. Both the CTEs during the heating (H) and cooling (C) processes were measured. Two specimens of each material were measured and the results indicated good reproducibility.

In terms of 3Y-TZP, the CTE values were between 1.0 × 10^−5^ /K and 1.2 × 10^−5^ /K, basically increasing or decreasing smoothly with respect to the temperature. Nevertheless, at a temperature higher than 1300 °C, the curve of length change was bent. One possible reason could be the occurrence of a slight tetragonal (T) to cubic (C) phase transformation, as reported in [30], where they found the redistribution of Y^3+^ ions at the grain boundary at 1300 °C, leading to the T (metastable) to C phase transformation. Nevertheless, since the time seems too short for the phase transformation, it is more likely that the ‘bend’ results from the creep behavior of the 3Y-TZP specimen at temperatures higher than 1300 °C, although a quite low compressive load of 0.25 N was applied.

**Figure 4:**
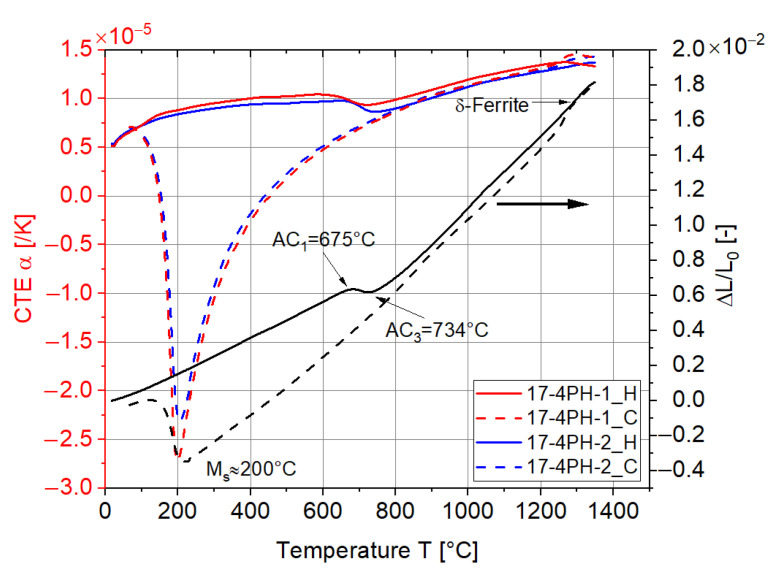
Length change and technical CTE results for 17-4PH during both heating and cooling stages.

In comparison to 3Y-TZP, 17-4PH had a much larger range of CTE values due to the different phase compositions resulting from the phase transformation, as shown in Figure 4. Moreover, the CTE values during cooling down process varied considerably from the heating up process. During heating, the temperature of 627 °C (AC_1_) indicated the occurrence of austenite (γ), while the temperature of 704 °C (AC_3_) implied the complete austenitization of 17-4PH. At a temperature higher than 1300 °C, the curve of the length change was bent slightly due to the formation of a small amount of δ-ferrite. During cooling, the CTE of 17-4PH decreased drastically due to the temperature at around 200 °C, where the martensitic transformation started. Below 200 °C, the austenite transformed into martensite, leading to the volume increase in the specimen. The significant drop in the CTE of 17-4PH during the cooling down process drew our attention, since the difference between the CTEs of 3Y-TZP and 17-4PH were quite large, especially at 200 °C. This may eventually result in delamination or failure if the interface between the two layers is not strong enough bonded.

### 2.4. Analytic Solution for the Curvature and Stress Distribution

**Figure 5:**
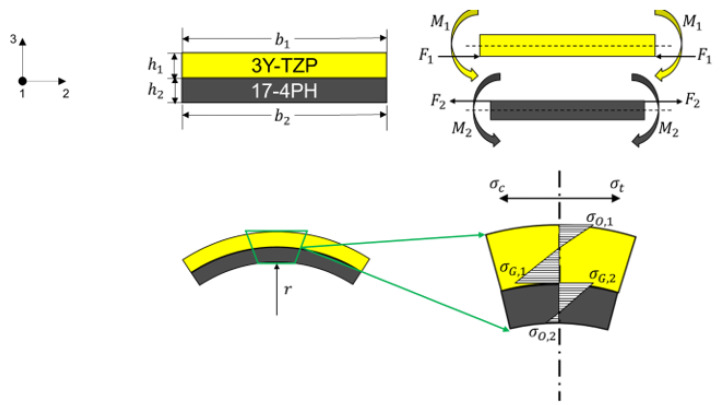
Schematic drawing of the curvature and stress distribution of a two-layer laminate and its coordinate system.

The analytic solution for two-layer MCLs is obtained based on the well-known Timoshenko beam theory [7,13]. The assumption of the analytic solution is the biaxial plane stress state, which indicates that the stresses in the two planar directions are identical. Based on this assumption and Hooke’s law, we may obtain:

(1) $$\varepsilon_{11}=\frac{1}{E}\left[\sigma_{11}-\nu \left(\sigma_{22}+\sigma_{33}\right)\right]=\frac{\left(1-\nu \right)}{E}\sigma_{11}=\frac{\sigma_{11}}{E^{\prime }}$$

where $E^{\prime }=E/\left(1-\nu \right)$, with E being Young’s modulus and ν being Poisson’s ratio. Here, $\varepsilon_{11}$ is the strain in 1 direction and $\sigma_{11}$, $\sigma_{22}$, and $\sigma_{33}$ are the stresses in 1, 2, and 3 directions, as shown in the coordinate system in Figure 5. The biaxial plane stress state is true if the planar sizes are similar in the two plane directions and much larger than the height (thickness) of the geometry, which was the case in this study, where the specimen had dimensions of 10 × 10 × 0.4 mm^3^.

As shown in Figure 5, provided the two layers are perfectly bonded and no delamination exists, then the radii r (curvature) of the two layers have to be identical:

(2) $$\frac{1}{r}=\frac{M_{1}}{E_{1}^{\prime }\cdot I_{1}}=\frac{M_{2}}{E_{2}^{\prime }\cdot I_{2}}$$

where M is the bending moment and $I=\frac{bh^{3}}{12}$ is the moment of inertia; b and h are the width and height of the corresponding layer, respectively (see Figure 5).

In addition, the strains at the interface of both layers ($\varepsilon_{1}$ and $\varepsilon_{2}$), which consist of strains due to force, the bending moment, and thermal expansion, have to be identical:

(3) $$\left\{\begin{array}{c} \varepsilon_{1}=-\frac{F_{1}}{E_{1}^{\prime }\cdot A_{1}}-\frac{M_{1}}{E_{1}^{\prime }\cdot I_{1}}\cdot \frac{h_{1}}{2}+\alpha_{1}\cdot \Delta T\\ \varepsilon_{2}=\frac{F_{2}}{E_{2}^{\prime }\cdot A_{2}}+\frac{M_{2}}{E_{2}^{\prime }\cdot I_{2}}\cdot \frac{h_{2}}{2}+\alpha_{2}\cdot \Delta T \end{array}\right.$$

where ΔT is the temperature change during the heating or cooling process. Moreover, the force at the end of interface of the two layers has to be equal. This yields:

(4) $$M=M_{1}+M_{2}=F\frac{h_{1}+h_{2}}{2}$$

where $F=F_{1}=F_{2}$.

Combining Equations (2)–(4), the normalized curvature of the two-layer MCLs can be analytically derived:

(5) $$k=\frac{h_{1}+h_{2}}{r}=\frac{6{\left(m+1\right)}^{2}mn_{e}\cdot \Delta T\left(\alpha_{1}-\alpha_{2}\right)}{m^{4}n_{e}^{2}+2mn_{e}\left(2m^{2}+3m+2\right)+1}$$

with$\;m=\frac{h_{1}}{h_{2}},\;n_{e}=\frac{E_{1}^{\prime }}{E_{2}^{\prime }}=\left(\frac{E_{1}}{1-\nu_{1}}\right)\left(\frac{1-\nu_{2}}{E_{2}}\right)$.

In addition to the curvature, the stress distribution of the two-layer MCLs can also be determined analytically. The stress is induced by the force and bending moment:

(6) $$\left\{\begin{array}{c} \sigma_{O,1}=-\frac{F}{A_{1}}+\frac{M_{1}}{I_{1}}\cdot \frac{h_{1}}{2}\\ \sigma_{G,1}=-\frac{F}{A_{1}}-\frac{M_{1}}{I_{1}}\cdot \frac{h_{1}}{2}\\ \sigma_{G,2}=\frac{F}{A_{2}}+\frac{M_{2}}{I_{2}}\cdot \frac{h_{2}}{2}\\ \sigma_{O,2}=\frac{F}{A_{2}}-\frac{M_{2}}{I_{2}}\cdot \frac{h_{2}}{2} \end{array}\right.$$

where $\sigma_{O,1}$ is the stress on the top side of the upper layer (3Y-TZP); $\sigma_{G,1}$ and $\sigma_{G,2}$ are respectively the stress at the interface of the upper layer and bottom layer (17-4PH); $\sigma_{O,2}$ is the stress on the downside of the bottom layer, as shown in Figure 5.

**Figure 6:**
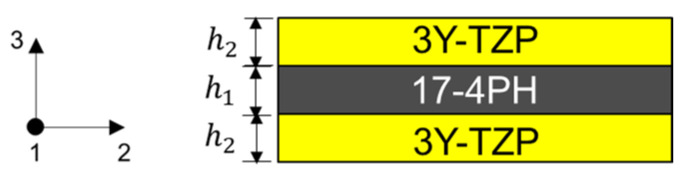
Schematic drawing of a three-layer laminate and its coordinate system.

In comparison to two-layer MCLs, the analytical solution of the symmetrical three-layer MCLs is easier to obtain thanks to the absence of curvature, as shown in Figure 6:

In the case of a perfectly rigid bonding between the layers, the strains of all layers have to be identical [31]:

(7) $$\frac{1-\nu_{1}}{E_{1}}\sigma_{11}^{1}+\alpha_{1}\Delta T=\frac{1-\nu_{2}}{E_{2}}\sigma_{11}^{2}+\alpha_{2}\Delta T$$

where $\sigma_{11}^{1}$ and $\sigma_{11}^{2}$ are the stress in 1 direction of components 1 and 2, respectively. In addition, the force equilibrium requires:

(8) $$\left(n-1\right)\sigma_{11}^{1}h_{1}+n\sigma_{11}^{2}h_{2}=0$$

where the symmetrical MCLs contains $\left(n-1\right)$ layers of component 1 and n layers of component 2. Combining Equations (7) and (8) gives us:

(9) $$\left\{\begin{array}{c} \sigma_{11}^{1}=-\Delta \varepsilon \frac{E_{1}^{\prime }}{1+\frac{\left(n-1\right)}{n}\frac{E_{1}^{\prime }}{E_{2}^{\prime }}\frac{h_{1}}{h_{2}}}\\ \sigma_{11}^{2}=\Delta \varepsilon \frac{E_{2}^{\prime }}{1+\frac{n}{\left(n-1\right)}\frac{E_{2}^{\prime }}{E_{1}^{\prime }}\frac{h_{2}}{h_{1}}} \end{array}\right.$$

with $E_{i}^{\prime }=\frac{E_{i}}{1-\nu_{i}};\;\Delta \varepsilon =\left(\alpha_{2}-\alpha_{1}\right)\Delta T$.

## 3. Results and Discussion for the Analytic Solution and FE Simulation

### 3.1. Comparison of the Analytic Solution and FE Simulation

The analytic solution and FE simulation results are compared in this section. For the two-layer MCLs, the analytic solution and FE simulation results for both the curvature and the stress distribution are compared. For the three-layer MCLs, only the average stress is compared, since no curvature exists in the analytic solution for symmetrical composites.

**Figure 7:**
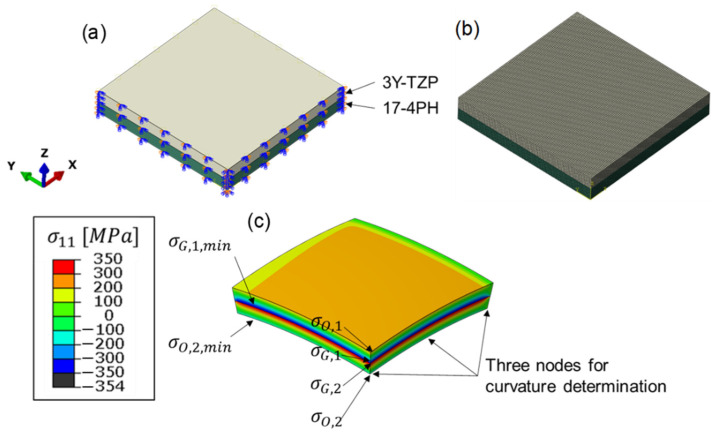
(**a**) The assembly of two-layer MCLs and boundary conditions. (**b**) Hexahedral mesh of laminates. (**c**) The curvature with a deformation scale factor of 10 and the stress distribution in the two-layer laminate.

Thanks to the symmetric structure, one-quarter of the two-layer MCLs were constructed in the FE simulation (ABAQUS 14-1) to reduce the computation time, as shown in Figure 7a. The input parameters for the Young’s moduli for 17-4PH and 3Y-TZP were firstly set as constant values at room temperature (see Figure 2). The corresponding Poisson’s ratio was calculated based on the measured Young’s moduli and shear moduli. The CTE of 17-4PH and 3Y-TZP were firstly assumed as 1.2 × 10^−5^ /K and 1.0 × 10^−5^ /K for this ideal scenario. The symmetric boundary conditions were set for the XZ- and YZ-plane. In addition, the interface between the two layers was perfectly tied as required by the analytic solution. The initial temperature of the two-layer MCLs was set to 1350 °C, and the MCLs were then cooled down simultaneously at a rate of 4 K/min to 25 °C. In other words, no temperature gradient inside the MCLs was considered here, as also required by the analytic solution. Afterwards, the two-layer MCLs were meshed with 160,000 C3D8T hexahedral elements, as shown in Figure 7b. C3D8T is an 8-node fully integrated thermal element constructed in Abaqus.

As a result of the simulation, the curvature and the stress distribution were obtained. The curvature of the FE simulation was obtained by exporting the coordinates of three nodes along the edge on the symmetric XZ-plane, as shown in Figure 7c. With the information for the coordinates changing according to the cooling time, the curvatures of the FE simulation were calculated with the help of Matlab 2021b. Apart from the curvatures, the time-dependent $\sigma_{11}$ values at the top side of the upper layer ($\sigma_{O,1}$), at the interface of the upper layer ($\sigma_{G,1}$) and bottom layer ($\sigma_{G,2}$), as well as on the down side of the bottom layer $\left(\sigma_{O,2}\right)$ were exported from the simulation. While $\sigma_{O,1}$ and $\sigma_{G,2}$ (tensile stresses) were always at the maximum, $\sigma_{G,1}$ and $\sigma_{O,2}$ (compressive stress) were not at the minimum in the structure. Therefore, the minimum stress $\sigma_{G,1,\;min}$ and $\sigma_{O,2,min}$ at corresponding nodes (see Figure 7c) were additionally exported for comparison.

**Figure 8:**
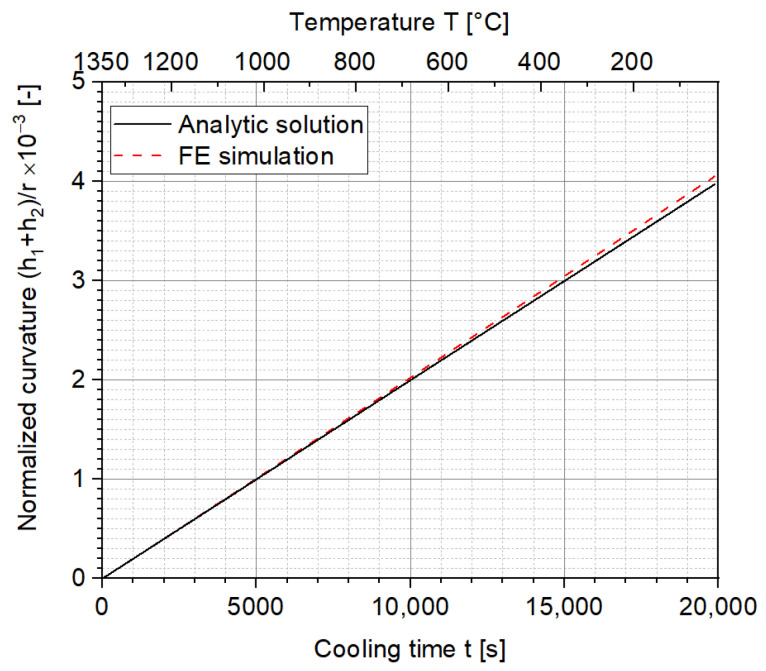
Comparison of the normalized curvatures between the analytic solution and FE simulation for the ideal scenario.

**Figure 9:**
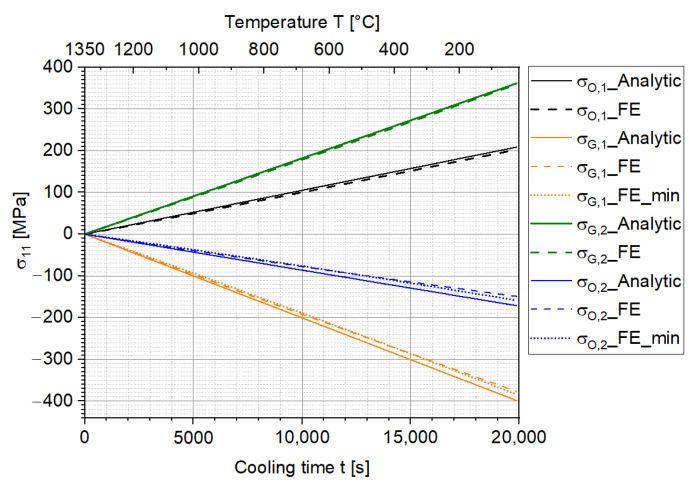
Comparison of the stress distributions in the two-layer MCLs between the analytic solution and FE simulation for the ideal scenario.

The analytic solution for the normalized curvature and the stress distribution results for the two-layer MCLs were respectively calculated according to Equations (5) and (6) with the help of Matlab 2021b. The results are shown in Figure 8 and Figure 9, where the normalized curvature and stress distribution between the analytic solution and FE simulation are compared. Since the relevant parameters (m, $n_{e}$, $\alpha_{1}$, and $\alpha_{2}$) were set constant in Equation (5) and ΔT is its only variable, which increases linearly according to cooling time, the analytic solution for the normalized curvature also increases linearly. Figure 8 shows a very good agreement (with a maximum difference of about 2%) between the analytic solution and FE simulation in terms of the normalized curvature in the two-layer MCLs.

According to Equation (4), the force F at the interface is proportional to the bending moment, which is proportional to the curvature, as indicated in Equation (2). Therefore, F changes linearly according to the curvature. Since the curvature changes linearly according to the cooling time, the stresses that are proportional to F in Equation (6) also change linearly based on the analytic solution.

The results are summarized and plotted in Figure 9. Again, the FE simulation results correspond well to the analytic solution in this ideal scenario. Nevertheless, the FE simulation slightly underestimates the stress values in all cases. Especially for $\sigma_{O,2}$, the maximum difference is up to 13%. This can be explained by the fact that the node stress values are obtained by extrapolating the integration point stresses based on the shape function. Since the integration points can neither be exactly located on the surface nor at the interface, numerical extrapolation errors may occur. It is believed that this numerical error can be further reduced by using a finer mesh. However, this is not included in the current study due to its high computational effort and time.

**Figure 10:**
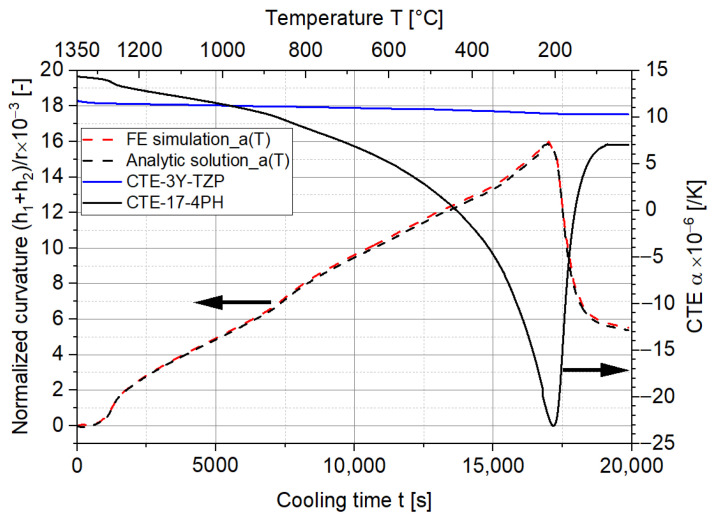
Comparison of the normalized curvatures between the analytic solution and FE simulation after consideration of the CTE during the cooling down process.

A further comparison was done by taking the temperature-dependent CTEs of 17-4PH and 3Y-TZP during cooling into account. The normalized curvatures and stresses were obtained following the same procedures as described for the ideal scenario. The CTEs of 17-4PH and 3Y-TZP for the cooling, analytical solution, and FE solution of curvatures are presented in Figure 10. In comparison to the ideal scenario, the normalized curvature is significantly larger due to the larger difference between the CTEs of the two materials. The maximum curvature occurs at around 200 °C (around 1% difference between the analytic solution and FE simulation), where the CTE difference is at the maximum and the martensitic transformation of 17-4PH starts to occur. As the transformation proceeds, the volume of 17-4PH increases, resulting in a smaller CTE difference as well as a lower curvature.

**Figure 11:**
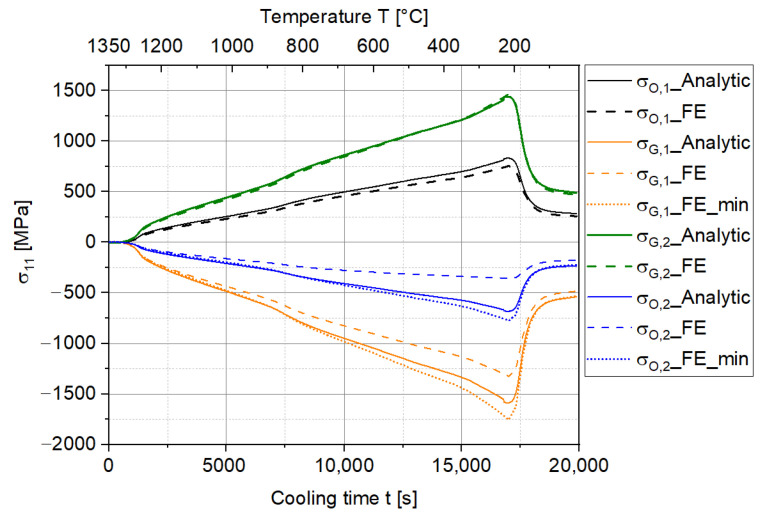
Comparison of the stress distributions of the two-layer MCLs between the analytic solution and FE simulation after consideration of the CTE during the cooling down process.

This lower curvature will relieve the stresses inside the MCLs, as shown in Figure 11. Despite the more complex evolution of the curvature and stresses in comparison to the ideal situation, the stress evolution in the FE simulation corresponds well to that in the analytic solution. Nonetheless, the maximum stress at the interface of the 17-4PH layer ($\sigma_{G,2}$) is close to 1.5 GPa, which will induce an immediate failure in the steel layer. This is not even close to reality, since the 17-4PH can deform plastically at high temperatures, which will eventually reduce the curvature and stress levels in the MCLs. This is out of the scope of the current study and will be investigated in the future after obtaining the stress–strain curve of 17-4PH at high temperatures.

**Figure 12:**
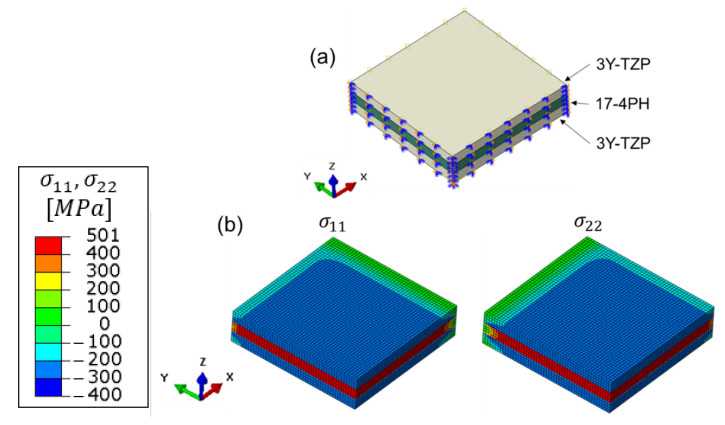
(**a**) The assembly of the three-layer MCLs and the boundary conditions. (**b**) The stress distribution of the three-layer MCLs based on the FE simulation.

In comparison to the two-layer MCLs, the analytic solution for the three-layer MCLs is simpler thanks to the absence of curvature. The FE simulation for the three-layer MCLs was carried out with constant E, ν $,\;\alpha_{1}$, and $\alpha_{2}$ values. The assembly and boundary of the three-layer MCLs are illustrated in Figure 12a, and the corresponding stress distribution is shown in Figure 12b. Due to the higher assumed constant CTE of 17-4PH, the middle layer (17-4PH) is in tension while the upper and bottom layers are in compression. The analytic solution for $\sigma_{11}$ in both layers was calculated based on Equation (9). Due to the assumption of the biaxial stress state in the analytic solution, $\sigma_{11}$ equals $\sigma_{22}$.

The average ${\bar{\sigma }}_{11}$ and ${\bar{\sigma }}_{22}$ values in the FE simulation were obtained by averaging the stress values at all integration points at the end of the cooling process. The results are summarized in Table 1. The stress values for the analytic solution and FE simulation were in good accordance and the difference was about 8%.

### 3.2. Influence of the E Modulus and Cohesive Contact

Until now, the Young’s moduli E and Poisson’s ratios ν of 17-4PH and 3Y-TZP at room temperature have been used for the determination of the curvature based on both the FE simulation and analytic solution. Nevertheless, E and ν depend on the temperature (see Figure 2). Thus, as indicated in Equation (5), the ratio $n_{e}$ of the temperature-dependent biaxial Young’s modulus $E^{\prime }=\frac{E}{1-\nu }$ also affects the curvature of the two-layer MCLs. This influence is investigated in this subsection.

**Figure 13:**
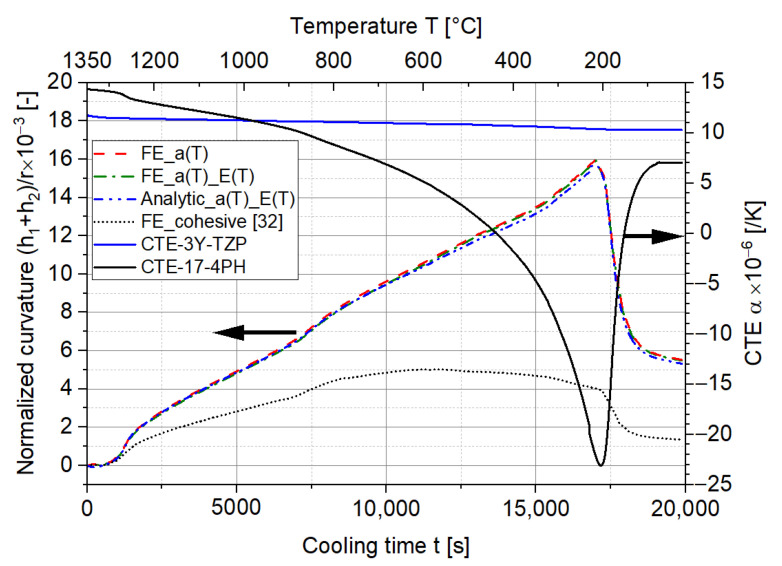
The influence of the temperature-dependent Young’s modulus and cohesive contact on the normalized curvature of the two-layer MCLs.

As can be observed in Figure 13, after considering the temperature-dependent $E^{\prime }$, the normalized curvature obtained from the FE simulation stays almost the same. The hardly observable difference indicates an insignificant influence of the ratio $n_{e}$ on the curvature. In addition, the analytic solution of the normalized curvature in this case is plotted. It corresponds very well to the FE simulation.

Furthermore, the influence of the interface was studied by defining the interface as a cohesive contact rather than a perfect tie in the FE simulation. The relevant parameters such as the stiffness, maximum traction stress, and fracture energy of the cohesive contact were adopted from [32] as a case study. In this respect, the energy of the interface is dissipated as the two-layer MCLs bend. Simultaneously, the dissipation of the energy reduces the bending and curvature of the MCLs considerably, as shown in Figure 13. This is an interesting point, since the significantly reduced curvature due to the energy consumption of the interface will eventually decrease the maximum stress existing in the MCLs, thereby improving the survival ratio during the cooling down process. Provided an ‘appropriate’ interface is formed between two layers, the failure could be avoided. For instance, an additional interlayer could be supplied to decrease the curvature as well as the stress level during the manufacturing process.

**Figure 14:**
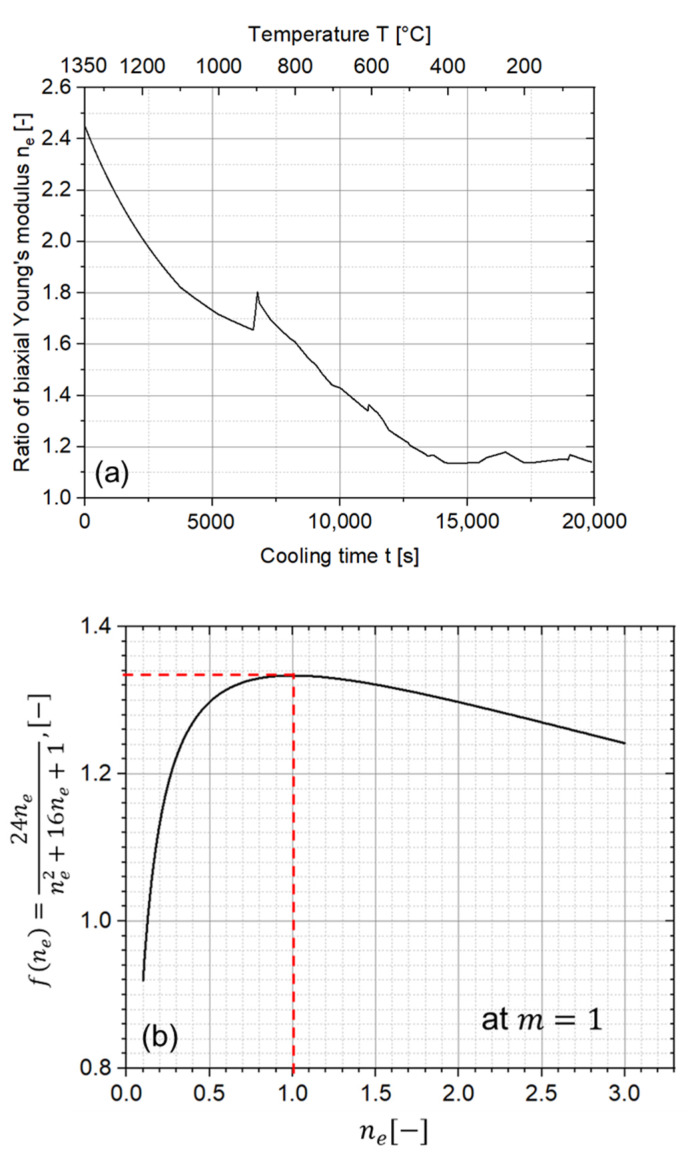
(**a**) The value of $n_{e}$ during cooling. (**b**) Influence of $n_{e}$ at m=1 based on Equation (5).

To facilitate a better understanding of the influence of $n_{e}$ on the curvature in terms of the analytic solution, the value of $n_{e}$ during the cooling down process is plotted in Figure 14a. In addition, the height ratio m is assumed to be one and the rest term of the $n_{e}$ values in Equation (5) are plotted in Figure 14b. Despite a slight increase at around 900 °C, the $n_{e}$ values generally decreases from 2.45 to 1.15 during the cooling down process, corresponding to an increase in $f\left(n_{e}\right)$ (see Figure 14b) from about 1.27 to 1.33. As mentioned in Section 3.1, the Young’s moduli were previously assumed as constant values at room temperature, where $n_{e}\;$equals 1.15 and $f\left(n_{e}\right)$ equals 1.33. After considering the temperature-dependent Young’s modulus, namely $n_{e}$, the value of $f\left(n_{e}\right)$ is always lower than 1.33, indicating a slight decrease in curvature. This can also be confirmed by the blue dashed line in Figure 13.

It can be further observed in Figure 14b that the most significant influence is found when $n_{e}$ equals one. In other words, the curvature is largest when the biaxial Young’s moduli $E^{\prime }$ of the two materials are the same. One may change $E^{\prime }$ in order to achieve a lower curvature. Nevertheless, this is only a theoretical perspective, since the E modulus is a mechanical property that usually depends not only on the porosity and temperature, but also on the chemical composition. Once the two materials are determined, the value of $n_{e}$ can hardly be altered.

### 3.3. Influence of Height Ratio

**Figure 15:**
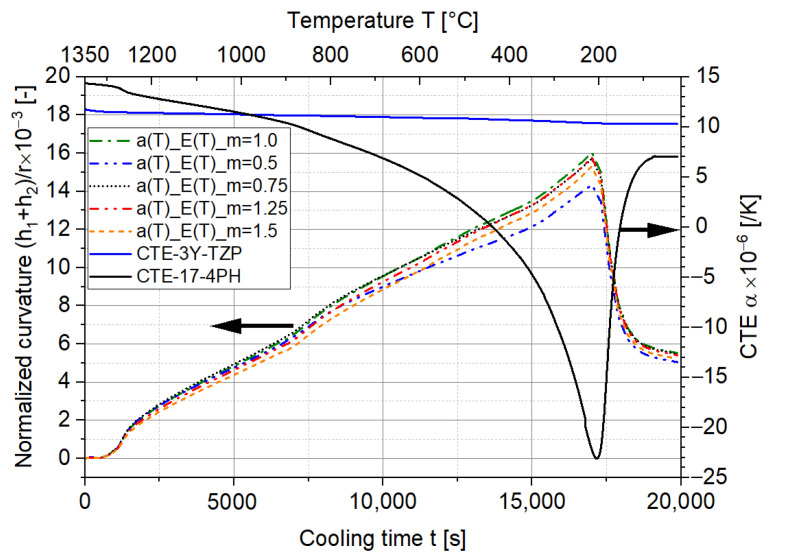
The influence of the height ratio on the normalized curvature of the two-layer MCLs based on the FE simulation.

In comparison to adjusting the material properties such as the Young’s moduli and CTEs of 17-4PH and 3Y-TZP, changing the height ratio seems to be a more feasible way to reduce the curvature and stress level in the two-layer MCLs. As plotted in Figure 15, the curvature varies with the different height ratios. While the largest curvature is found when the two layers have the same height (m=1), the lowest is found when one layer has a much higher thickness than the other.

**Figure 16:**
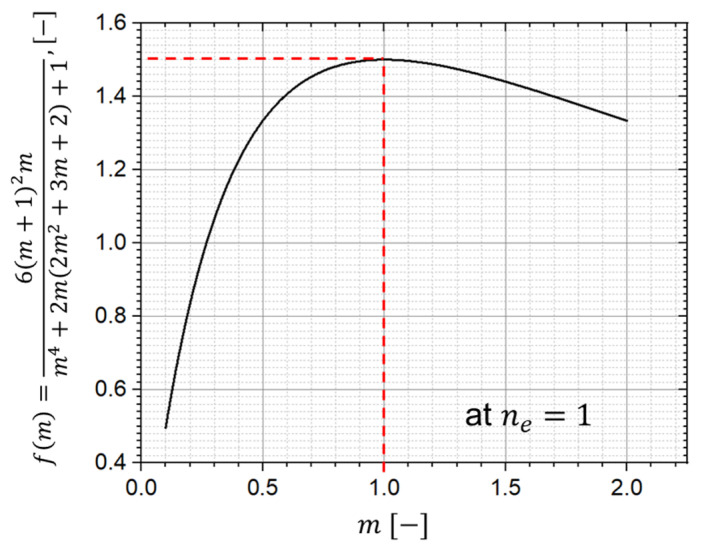
Influence of m at $n_{e}=1$ according to Equation (5).

A similar influence can be found for the analytical solution, and the term of variable m of Equation (5) at $n_{e}=1$ is plotted in Figure 16. The largest contribution of m to the curvature is found when two layers of the same height are constructed. Increasing or decreasing the value of m will reduce the maximum curvature. In addition, the curvatures at m=0.5 and m=2 are identical due to symmetry. This indicates that a lower curvature and stress level can be achieved by making one layer considerably thicker or thinner than the other. However, this is also technically challenging, since the maximum and minimum cast height of a single tape are strictly restricted by a lot of factors, such as the particle size, viscosity of the suspension, and others. Additionally, both very thick and very thin layers are expected to experience higher anisotropic sintering shrinkage due to the higher inhomogeneous relative density distribution during the tape casting. This high anisotropy may already lead to fractures during heating in the co-sintering process. Therefore, the sintering simulation has to be additionally carried out to find the best trade-off involving different influencing factors.

## 4. Conclusions

- The FE simulation of the curvature of the two-layer MCLs corresponds very well to the analytic solution. Meanwhile, the stress values are generally slightly lower than the analytic solution due to the numerical extrapolation error in the FE simulation.
- The CTE differences have the most significant influence on the curvature and stress distribution of the laminates, whereas the influence of the change in the elastic constants with temperature for the investigated material combination is less pronounced.
- The height ratio also affects the curvature of the two-layer MCLs, and lower curvatures occur when one layer has a much higher or lower thickness than the other.
- The cohesive contact reduces the curvature of the two-layer MCLs considerably due to the energy dissipation mechanism. This provides hints to avoid delamination during the manufacturing of the laminates.

It is noted that the above conclusions depend on the MCLs being free of stress and curvature at the beginning of the cooling down step. The different sintering shrinkage rates of 3Y-TZP and 17-4PH during the heating and dwell periods were not considered. Additionally, the plastic strain of the steel layer resulting from yielding at high temperatures, the creep, and the transformation plasticity is neglected. All of these aspects have to be studied in order to predict the residual stress and curvature of the MCLs numerically. These aspects will be addressed in future work.

## Figures and Tables

**Table 1:** Comparison of the stresses in the three-layer MCLs between the analytic solution and FE simulation.

Materials	$\mathrm{Analytic}\;\mathrm{Solution}\;\sigma_{11}\;[\mathrm{MPa}]$	FE Simulation	
		${\bar{\sigma }}_{11}\;[\mathrm{MPa}]$	${\bar{\sigma }}_{22}\;[\mathrm{MPa}]$
3Y-TZP	−247	−227	−227
17-4PH	494	456	456

## Data Availability

The data presented in this study are available on request from the corresponding author.

## Nomenclature

CTE	Coefficient of thermal expansion	b	Width of the cross-section
MCLs	Metal–ceramic laminates	h	Height of the cross-section
TB	Timoshenko beam	r	Radius of the curvature
VE	Viscous-elastic	A	Area of the cross-section
SOFC	Solid oxide fuel cell	F	Force
FE	Finite element	k	Normalized curvature
σ	Stress	m	Layer height ratio
ε	Strain	ΔT	Temperature difference
E	Young’s modulus	α	Coefficient of thermal expansion
G	Shear modulus	$\sigma_{O,1}$	Stress on the top side of the upper layer
ν	Poisson’s ratio	$\sigma_{G,1}$	Stress at the interface of the upper layer
$E^{\prime }$	Biaxial Young’s modulus	$\sigma_{G,2}$	Stress at the interface of the bottom layer
$n_{e}$	Ratio of biaxial Young’s modulus	$\sigma_{O,2}$	Stress on the downside of the bottom layer
M	Bending moment	n	Number of layers
I	Moment of inertia		
